# Genomic insight into diet adaptation in the biological control agent *Cryptolaemus montrouzieri*

**DOI:** 10.1186/s12864-021-07442-3

**Published:** 2021-02-25

**Authors:** Hao-Sen Li, Yu-Hao Huang, Mei-Lan Chen, Zhan Ren, Bo-Yuan Qiu, Patrick De Clercq, Gerald Heckel, Hong Pang

**Affiliations:** 1 State Key Laboratory of Biocontrol, School of Life Sciences / School of Ecology, Sun Yat-sen University, Guangzhou, Guangdong China; 2 School of Environment and Life Science, Nanning Normal University, Nanning, 530001 China; 3 Department of Plants and Crops, Faculty of Bioscience Engineering, Ghent University, Ghent, Belgium; 4 Institute of Ecology and Evolution, University of Bern, Bern, Switzerland

**Keywords:** Genome, Biological control, Ladybird, *Cryptolaemus montrouzieri*, Prey adaptation, Immunity, Evolution

## Abstract

**Background:**

The ladybird beetle *Cryptolaemus montrouzieri* Mulsant, 1853 (Coleoptera, Coccinellidae) is used worldwide as a biological control agent. It is a predator of various mealybug pests, but it also feeds on alternative prey and can be reared on artificial diets. Relatively little is known about the underlying genetic adaptations of its feeding habits.

**Results:**

We report the first high-quality genome sequence for *C. montrouzieri*. We found that the gene families encoding chemosensors and digestive and detoxifying enzymes among others were significantly expanded or contracted in *C. montrouzieri* in comparison to published genomes of other beetles. Comparisons of diet-specific larval development, survival and transcriptome profiling demonstrated that differentially expressed genes on unnatural diets as compared to natural prey were enriched in pathways of nutrient metabolism, indicating that the lower performance on the tested diets was caused by nutritional deficiencies. Remarkably, the *C. montrouzieri* genome also showed a significant expansion in an immune effector gene family. Some of the immune effector genes were dramatically downregulated when larvae were fed unnatural diets.

**Conclusion:**

We suggest that the evolution of genes related to chemosensing, digestion, and detoxification but also immunity might be associated with diet adaptation of an insect predator. These findings help explain why this predatory ladybird has become a successful biological control agent and will enable the optimization of its mass rearing and use in biological control programs.

**Supplementary Information:**

The online version contains supplementary material available at 10.1186/s12864-021-07442-3.

## Background

The remarkable evolutionary success of insects is associated with adaptations to a vast diversity of food sources and access to multiple trophic niches. For example, the emergence of gene families encoding odorant binding proteins and odorant receptors allowed insects to locate new diet sources [1]. The expansion of diet range is associated with the expansion of gene families related to detoxification and digestion [2, 3]. In beetles, several studies have demonstrated that the adaptation to plant feeding includes the evolution of genes encoding chemosensors for finding appropriate food sources [4], digestive enzymes for breaking down plant cell walls [5–7], and detoxifying enzymes for eliminating harmful plant toxins [7, 8]. In addition, diet also affects insect immunity [9]. The evolution of insect immunity allows insects to change their phenotype in response to changes in the environment, including diet and microbiota [10]. For example, insect antimicrobial peptides (AMPs) can maintain a core microbiota while protecting against microbes [11]. In ladybird beetles, the invasive species *Harmonia axyridis* (Pallas, 1773) has more genes encoding AMPs than non-invasive species, which might reflect its invasive biology [12]. However, it is not clear whether feeding related traits, e.g. predatory efficiency, prey specialization and adaptability to feeding on unnatural or artificial foods in some beetle species is associated with similar patterns of genomic evolution, while these traits are of particular importance in biological control use.

The use of predatory insects in classical and augmentative biological control programs has yielded in some cases huge economic and ecological benefits. After the successful control of cottony cushion scales using the vedalia ladybird beetle *Novius cardinalis* (Mulsant, 1850) [13] in California in 1888–1889, hundreds of predatory insects were introduced from abroad for biological control purposes all around the world, but most of them failed to establish or provide pest control [14]. Some species used in classical or augmentative biological control programs even became invasive and harmed local biodiversity [15]. In contrast, the mealybug destroyer *Cryptolaemus montrouzieri* Mulsant, 1853 is a successful predator and is still being used worldwide [16]. This predatory ladybird beetle is native to Australia and has been introduced to at least 64 countries or regions for classical or augmentative biological control purposes since 1891 [16]. The success of *C. montrouzieri* can be attributed to its efficient predation of mealybug pests and easy mass rearing [16–18].

Mealybugs (Hemiptera, Sternorrhyncha, Pseudococcidae) are the predominant prey of *C. montrouzieri*. Whereas mealybugs produce wax secretions to protect themselves from a range of natural enemies, these wax secretions act as an attractant and oviposition stimulant for *C. montrouzieri* [16], indicating ladybird-mealybug specialization. Under laboratory and mass rearing conditions, *C. montrouzieri* can also feed on other Sternorrhyncha species (e.g., whiteflies, aphids and other coccids), lepidopteran eggs and even artificial diets [18–21]. Some of these alternative diets can support the complete life cycle of the ladybird (provided that an artificial oviposition substrate is supplied) but will to some extent decrease fitness of the predators. Previously, we detected a large number of differentially expressed genes (DEGs) in *C. montrouzieri* in response to a diet shift from mealybugs to aphids [21]. This suggests that *C. montrouzieri* can adapt to a variety of nutritional conditions via phenotypic and transcriptional plasticity.

**Fig. 1:**
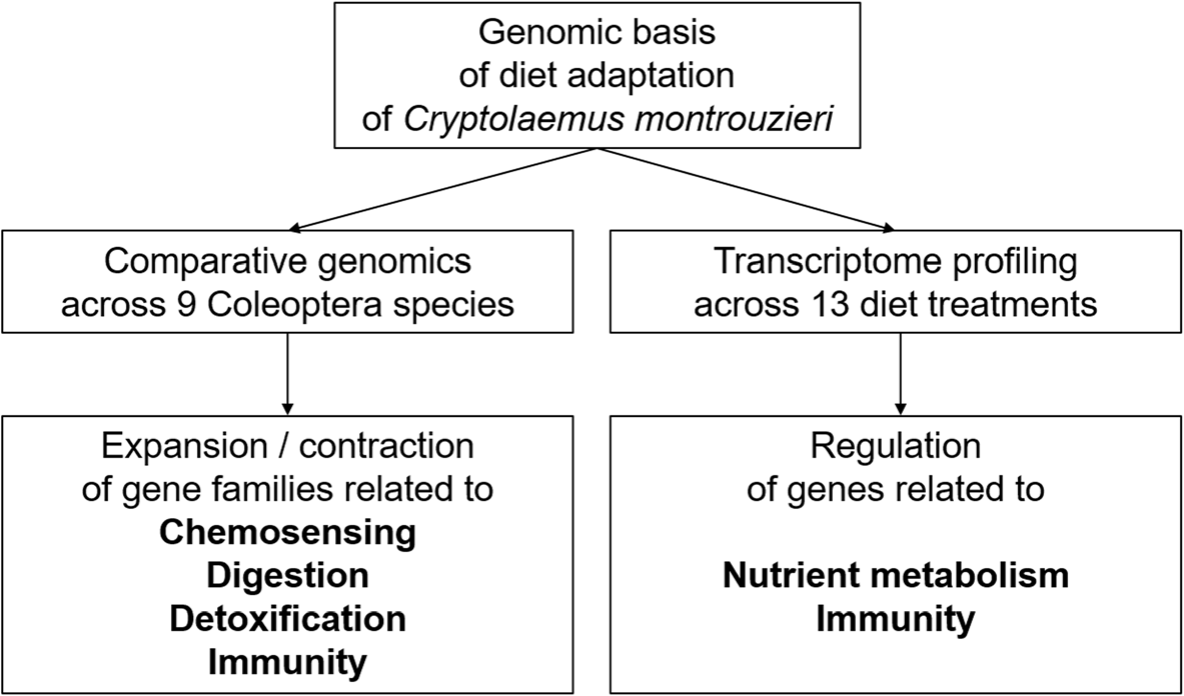
The study design for exploring the genomic basis of diet adaptation of *Cryptolaemus montrouzieri*

In this study, we hypothesize that diet adaptation of *C. montrouzieri* is associated with evolution and regulation of genes related to chemosensing, digestion, detoxification and immunity. We used genomic and transcriptomic approaches to examine the extent of dietary adaptation in *C. montrouzieri* (Fig. 1). We assembled a high-quality genome of *C. montrouzieri* and compared its content to eight other Coleoptera genomes. We further tested for gene expression differences between *C. montrouzieri* larvae that were experimentally fed different diets.

## Results

### General genomic features of *C. montrouzieri*

A total of 115.55 Gb of raw data and 106.63 Gb of high-quality clean reads were generated with PromethION DNA sequencing (Oxford Nanopore, UK). These Nanopore data together with additional 151.03 Gb Illumina data were assembled using Wtdbg, followed by Racon and Pilon polishing, which produced a 988.11 Mb genome assembly with 398 contigs and a contig N50 of 9.22 Mb (shortest: 39,165 bp; longest: 32,637,267 bp). This genome size of *C. montrouzieri* was larger than that of published ladybird and other Coleoptera genomes (largest among the ladybirds, *Propylea japonica* (Thunberg, 1781), 850.90 Mb; largest among Coleoptera, *Anoplophora glabripennis* (Motschulsky, 1853), 981.42 Mb) [5, 22]. Application of the Benchmarking Universal Single-Copy Orthologs (BUSCO, Insecta set) pipeline [23] showed that this *C. montrouzieri* genome compared well with the other insect genomes in the OrthoDB v10.1 database in terms of completeness, with 97.1% complete genes (96.0% single copy and 1.1% duplicated), 0.7% fragmented and 2.2% missing at the genome level.

Annotation of the *C. montrouzieri* genome using the Braker pipeline [24] yielded a final set of 27,858 genes and 32,187 protein sequences. Application of the BUSCO pipeline showed that this *C. montrouzieri* gene set has 93.1% complete genes (91.9% single copy and 1.2% duplicated), 4.2% duplicated and 2.7% missing at the protein level in the Insecta of OrthoDB database. In the functional annotation of this protein set, 31,632 were found in the National Center for Biotechnology Information (NCBI) nonredundant (NR) Hexapoda subset, 30,884 in Swiss-Prot, 16,042 in at least one protein domain in Pfam, 7613 in Gene Ontology (GO) and 8290 in Kyoto Encyclopedia of Genes and Genomes (KEGG) databases (Additional file 2: Table S1).

### Comparative genomics

**Fig. 2:**
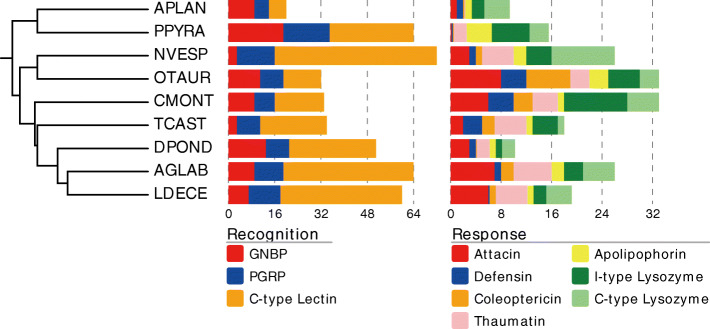
Evolution of immunity-related genes in *Cryptolaemus montrouzieri*. Number of genes related to immune recognition and response identified from nine beetle genomes. The species’ ultrametric tree was adapted from Mckenna et al. [6]. Each term contains genes that produce the same protein. Abbreviations of the tested species can be found in Table 1

A genome-wide scan of gene family evolution was performed among the genomes of *C. montrouzieri* and eight other Coleoptera species with different feeding habits (Table 1) [4, 5, 25–29]. As revealed by the clustering algorithm implemented in CAFE software [30], we found that 2426 and 2577 gene families of the *C. montrouzieri* genome underwent expansion and contraction, respectively (Additional file 1: Fig. S1). Among these, only 28 gene families underwent significant contraction (*P* < 0.05), among which two encode chemosensors and eight encode digestive or detoxifying enzymes (Table 2 and details in Additional file 2: Table S2). Of the 598 significantly expanded gene families in *C. montrouzieri* (*P* < 0.05), one encodes chemosensors, nine encode digestive or detoxifying enzymes, and one encodes immune effectors (Table 2 and details in Additional file 2: Table S2). Further identification of immunity-related genes showed that *C. montrouzieri* had a large number of genes encoding immune effectors including 18 antimicrobial peptides (AMPs) and 15 lysozymes. This number of immune effector genes is equal to those of the beetle *Onthophagus taurus* (Schreber, 1759) [25] and larger than the other beetles (Fig. 2). In contrast, only 33 genes of *C. montrouzieri* are involved in recognition, while some of the other beetles have around 60 of these genes (Fig. 2).

**Table 1:** Genomes of Coleoptera species with different feeding habits used for comparative genomic analyses. Species IDs were ordered based on the species tree topology (Additional file 1: Fig. S1)

Species ID	Species	Family	Feeding habit	Reference
APLAN	*Agrilus planipennis* Fairmaire, 1888	Buprestidae	Herbivorous	[25]
PPYRA	*Photinus pyralis* (Linnaeus, 1767)	Lampyridae	Carnivorous in larva stage	[26]
NVESP	*Nicrophorus vespilloides* Herbst, 1783	Staphylinidae	Saprophagous	[27]
OTAUR	*Onthophagus taurus* (Schreber, 1759)	Scarabaeidae	Saprophagous	[25]
CMONT	*Cryptolaemus montrouzieri* Mulsant, 1850	Coccinellidae	Carnivorous	This study
TCAST	*Tribolium castaneum* (Herbst, 1797)	Tenebrionidae	Herbivorous	[28]
DPOND	*Dendroctonus ponderosae* (Hopkins, 1902)	Curculionidae	Herbivorous	[29]
AGLAB	*Anoplophora glabripennis* (Motschulsky, 1854)	Cerambycidae	Herbivorous	[5]
LDECE	*Leptinotarsa decemlineata* Say, 1824	Chrysomelidae	Herbivorous	[4]

**Table 2:** Significant expansion (E) or contraction (C) of *Cryptolaemus montrouzieri* in gene families encoding chemosensor, digestive and detoxifying enzymes and immunity as detected with CAFE in comparison to the other beetle genomes. Orthologous genes were identified by Orthofinder. The number of genes in each family are shown for each species. These gene families contain protein domain of: odorant receptors (OR), odorant binding protein (OBP), maltase, glycosyl hydrolase (GH), trypsin, cathepsin, cytochrome P450 (P450), UDP-glucuronosyltransferases (UGT), carboxylesterase (CE) and attacin. Abbreviations of the tested species are defined in Table 1

Function	Gene family	E/C	APLAN	PPYRA	NVESP	OTAUR	CMONT	TCAST	DPOND	AGLAB	LDECE
Chemosensor	OR	C	0	0	4	3	**0**	18	5	15	12
	OR	C	9	1	5	2	**0**	0	1	3	6
	OBP	C	4	3	19	14	**2**	15	7	18	6
	OBP	E	0	0	0	0	**5**	0	0	0	2
Digestive enzyme	Maltase	E	20	2	0	2	**5**	2	1	3	1
	GH1	C	23	8	6	5	**6**	13	19	22	26
	GH16	E	4	0	1	1	**5**	1	10	2	3
	Trypsin	C	30	4	2	17	**3**	4	14	22	4
	Trypsin	C	5	39	26	19	**5**	6	14	20	5
	Trypsin	C	26	2	2	6	**0**	1	4	10	1
	Trypsin	C	2	4	5	2	**0**	2	0	3	7
	Cathepsin L	E	0	0	0	0	**20**	0	7	10	24
	Cathepsin B	E	1	1	1	1	**6**	2	1	1	14
Detoxifying enzyme	P450	C	16	22	2	18	**7**	34	3	27	7
	P450	E	3	4	1	4	**9**	3	2	12	4
	UGT	C	6	12	3	1	**3**	25	7	10	8
	UGT	C	0	8	5	9	**0**	3	1	4	3
	UGT	E	0	1	0	2	**10**	0	0	0	2
	UGT	E	5	0	0	2	**5**	3	1	0	0
	UGT	E	0	0	0	0	**11**	0	0	0	0
	CE	E	1	25	5	1	**7**	1	1	1	1
Immunity	Attacin	E	0	0	1	5	**7**	2	1	6	5

### Feeding experiment and transcriptome profiling

The responses of both life history traits and gene expression to different diets were experimentally studied. Second-instar *C. montrouzieri* larvae were raised on 13 diets, including one natural prey diet and 12 factitious prey or artificial diets (Table 3). The natural prey *Planococcus citri* (Risso, 1813) (MEALYBUG) has been used to maintain the tested laboratory population for more than 10 years. Thus, the use of this prey for *C. montrouzieri* as a control allows comparison of the responses to different diets.

**Table 3:** Diet design for *Cryptolaemus montrouzieri* larvae

Diet type	Protein sources	Processing	Code
Invertebrate whole bodies	Mealybug *Planococcus citri* (Risso, 1813)	Live prey	MEALYBUG
	Pea aphid *Megoura japonica* (Matsumura)	Live prey	PEAAPHID
	Larvae of yellow mealworm *Tenebrio molitor* Linnaeus, 1758	Dry powder and solid medium	MEALWORM
	Larvae of house fly *Musca domestica* Linnaeus, 1758	Dry powder and solid medium	HOUSEFLY
	Earthworms	Dry powder and solid medium	EARTHWORM
	Pupae of honeybee *Apis mellifera* Linnaeus, 1758	Dry powder and solid medium	HONEYBEE
	Larvae of black soldier fly *Hermetia illucens* Linnaeus, 1758	Dry powder and solid medium	SOLDIERFLY
Invertebrate eggs	Eggs of flour moth *Ephestia cautella* (Walker, 1863)	Frozen	FLOURMOTH
	Eggs of rice moth *Corcyra cephalonica* (Stainton, 1866)	Frozen	RICEMOTH
	Cysts of brine shrimp *Artemia salina* (Linnaeus, 1758)	Medium	BRINESHRIMP
Vertebrate materials	Pork liver	Dry powder and solid medium	PORKLIVER
	Chicken egg	Solid medium	CHICKENEGG
Plant materials	Pollen of *Brassica campestris* Linnaeus	Solid medium	POLLEN

**Fig. 3:**
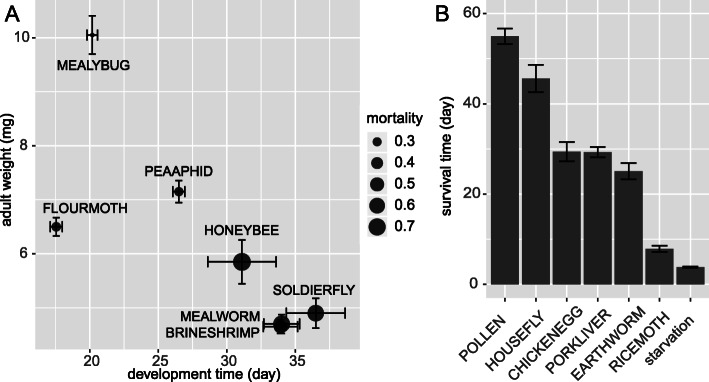
Comparison of life history traits of *C. montrouzieri* fed different diets. **a** Effect of different diets on the development, adult weight and mortality of *C. montrouzieri* larvae. Error bars show the standard deviation. **b** Survival time of larvae with different diets that did not allow development to the adult stage

In the comparison of life history traits, we found large differences in the performance of the larvae among these 13 diets (Fig. 3 and details in Additional file 1: Table S3). The natural prey diet (MEALYBUG) was clearly favorable, with the highest adult weight, second shortest development time and lowest mortality rate (Fig. 3a). The two factitious prey diets PEAAPHID and FLOURMOTH were second only to the natural prey diet in terms of adult weight. Individuals in the remaining ten diet treatments performed much worse, with > 70% mortality or failure to develop to the adult stage on six of those diets. The 12 unnatural diets overall led to significantly longer larval survival than no food (Fig. 3b), especially the POLLEN diet, which sustained larvae for up to 50 days.

**Fig. 4:**
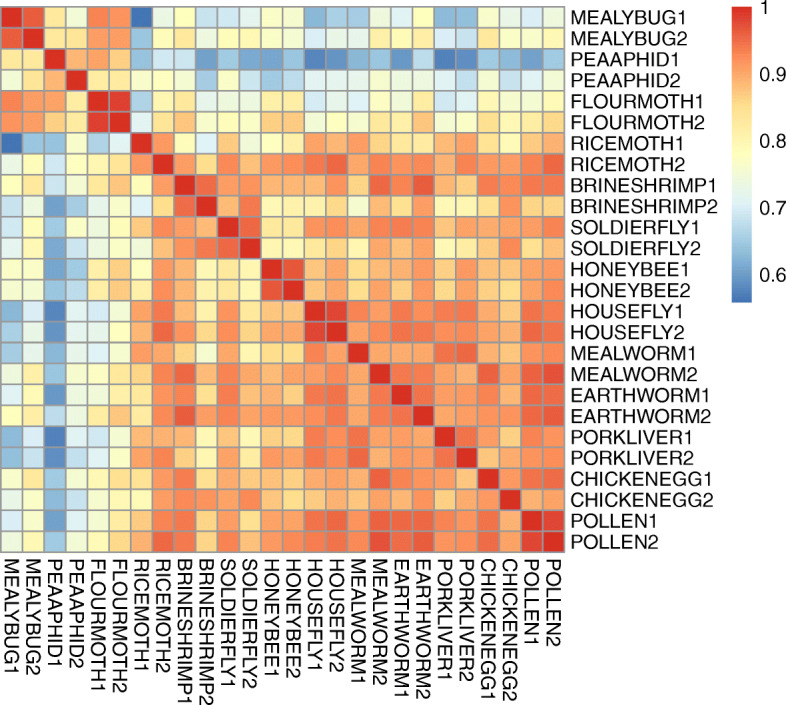
Relationship (*r*^2^) of gene expression between the studied transcriptomes with different diet treatments. Abbreviations of diet treatments can be found in Table 3

**Fig. 5:**
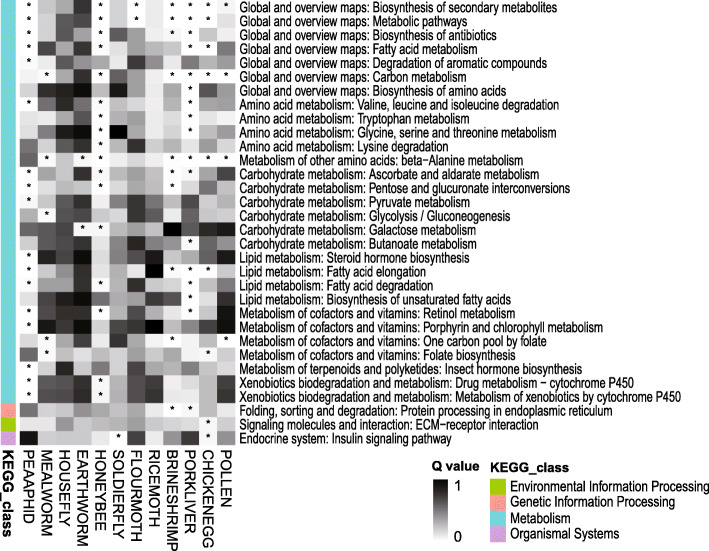
Heatmap of adjusted P values (Q) in KEGG pathway enrichment analysis for the transcriptome comparisons of alternative diets versus the natural prey of *C. montrouzieri* larvae. Enrichment with Q < 0.05 is marked with an asterisk. Twenty-nine out of 32 enriched pathways were related to metabolism

Gene expression was then profiled in 4th-instar larvae (< 24 h after molting) fed different diets. An overview of relative gene expression levels in the 13 treatments is presented by a heatmap of *r*^2^ values in Fig. 4. All of the treatments had *r*^2^ values between the two replicates exceeding 0.88, indicating repeatability within treatments. The top three favorable treatments in life history trait comparisons (MEALYBUG, PEAAPHID and FLOURMOTH) shared also high *r*^2^ values among each other (0.82–0.93, mean = 0.88). Gene expression patterns in these three treatments were usually more different from the inferior diet treatments (*r*^2^: 0.56–0.88, mean = 0.73). When comparing the 12 unnatural (i.e. factitious prey or artificial diet) treatments with the MEALYBUG treatment, DEGs were enriched in 32 KEGG pathways (Q < 0.05), among which 29 were related to nutrient or toxin metabolism (Fig. 5). Similarly, the DEGs were enriched in GO terms mainly related to nutrient metabolism processes and catalytic/oxidoreductase activities (Q < 0.05, Additional file 1: Fig. S2).

**Fig. 6:**
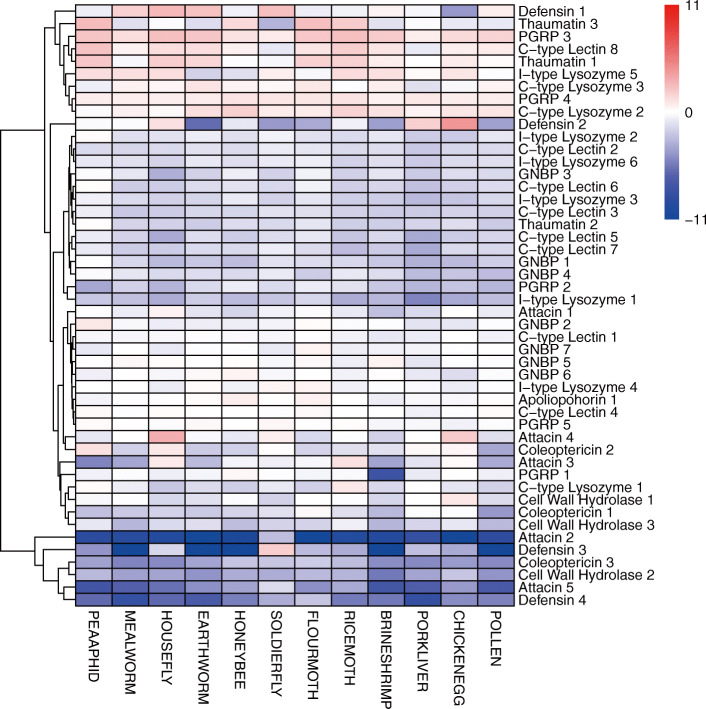
Transcriptome profiling of *C. montrouzieri* larvae fed different diets. Heatmap shows the log2(fold change) for the transcriptome comparisons of alternative diets versus the natural prey of *C. montrouzieri*. Hierarchical clustering of the heatmap was carried out using pheatmap package in R. Only genes related to immune recognition and response are shown. Abbreviations of diet treatments can be found in Table 3. PGRP: peptidoglycan recognition protein. GNBP: gram-negative binding protein

As we found a significant expansion in a gene family involved in the immune response in *C. montrouzieri* (Table 2), the pattern of expression of immune effector genes including those encoding antibacterial peptides (AMPs) and lysozymes was specifically analyzed. We found a general down regulation of the immune effector genes (log2-fold change mean ± SE: − 1.73 ± 0.15) as compared to the natural prey MEALYBUG treatment. Among them, 6 of 28 were dramatically downregulated when larvae shifted their diet from mealybugs to unnatural diets, with most of the log2-fold change values lower than − 3 (i.e. nine times lower than those in the natural prey control, Fig. 6). These genes include two *attacin* genes, two *defensin* genes, one *coleoptericin* gene and one *cwh* gene. In contrast, only slight regulation of expression (log2-fold change mean ± SE: − 0.69 ± 0.08) was detected in genes related to immune recognition including those encoding c-type lectin, peptidoglycan recognition protein (PGRP) and gram-negative binding protein (GNBP) (Fig. 6).

## Discussion

### Gene expansion/contraction related to feeding habits

The order Coleoptera is the most speciose group of animals with highly diverse feeding habits. Most of the species in the suborder Adephaga are predaceous while Polyphaga (e.g. weevils, longhorn beetles and leaf beetles) are predominantly phytophagous species. The high diversity of phytophagous beetles can be explained by their complex interactions with flowering plants [8, 31, 32]. However, Polyphaga also includes the ladybird beetles (Coccinellidae), most of which are predaceous [33]. Evolutionary studies have suggested that the ancestral ladybirds have switched from mycophagy to a predatory life style [33–35]. This is associated with the diversification of ladybirds into more than 6000 reported species [34].

In this study, we explored the evolutionary patterns of gene families involved in the functions of chemosensing, digestion, detoxification and immunity in the predatory ladybird *C. montrouzieri* as compared with other beetles with different feeding habits. We found that the *C. montrouzieri* genome has undergone significant expansion or contraction of several gene families encoding chemosensors, digestive and detoxification enzymes. It seems that these gene families are usually involved in diet adaptation of not only phytophagous but also predatory beetles. The evolution of these gene families of *C. montrouzieri* might be associated with adaptation to mealybug feeding. However, we also need to be aware of the other potential factors (e.g. the distinct phylogenetic relationship of the tested species, the different qualities of genome assemblies and annotations) that affect these patterns of genome evolution.

### Deficiencies of novel diets and diet-induced gene regulation

The availability of a cost-effective factitious prey or artificial diet is key to the successful mass production of arthropod natural enemies for use in biological control [36]. The availability of molecular markers that could be used as early indicators of insect responses to general and specific nutritional levels may greatly advance the practice of insect mass rearing [37–40]. However, very few biological control agent species have completely sequenced genomes. In this study, with the help of our new completely sequenced *C. montrouzieri* genome, diet-induced gene regulation was explored, which might further benefit diet development. We found that the performance of *C. montrouzieri* larvae fed unnatural diets decreased to different degrees. Both life history trait performance and the pattern of gene expression congruently revealed that the feeding treatments with another Sternorrhyncha species (PEAAPHID) or lepidopteran eggs (FLOURMOTH) were relatively close to those with natural prey. In comparison, individuals in the rest of the treatments performed much worse and had more divergent patterns of gene expression. DEGs in the unnatural diet treatments compared to the natural prey treatment were mainly enriched in nutrient metabolism. This finding explores a relationship between diet and regulation of gene expression, and suggests that the decrease in performance might be caused by a dietary nutrient imbalance. Several studies have tried to explore the relationship between gene expression patterns and diet limitation of predatory biological control agents, and have tried to further optimize the diet formulations [37–41]. However, the mechanism of how diet components affect gene expression of these predators is still not clear. Similarly, our current data are insufficient to pin down the exact deficiencies of the tested diets. More detailed studies are needed to explore the relationship between diet components and gene expression.

### Potential roles of immune enhancement in prey adaptation

Previous studies have demonstrated that many genes encoding AMPs and lysozymes of ladybirds are dramatically upregulated under the challenge of bacterial infection [12, 42]. In our study, the expression of some genes encoding AMPs and cell wall hydrolases was dramatically downregulated when using unnatural diets. Change of nutritional condition is known to cause significant changes in the physiology of ladybirds [43] and other predatory insects [44]. Thus, it is possible that using an unnatural diet has a negative effect on the physiology of ladybirds, including immune defense. Several studies have demonstrated the impact of diet on insect immune response [45–47]. However, their findings show that diet changes can positively impact on certain immune traits but negatively affect others. In addition, little evidence in insects supports a relationship between diet changes and downregulation of immune effector genes [48]. Alternatively, it seems that using natural prey, i.e. mealybugs, is one of the factors inducing immune responses in *C. montrouzieri*. Furthermore, the gene family encoding attacin has expanded in comparison to other Coleoptera genomes. These two pieces of evidence together suggest a potential role of immune enhancement in prey adaptation of *C. montrouzieri*.

The main prey of predatory ladybirds include aphids, coccids (e.g., mealybugs and scale insects), psyllids and whiteflies, all of which are in the suborder Sternorrhyncha (Hemiptera). These sap-feeding insects have a specific bacteriome that harbors a large number of symbiont bacteria [49]. It is possible that the symbiont bacteria of mealybugs cause an immune response in ladybird predators. A growing body of evidence shows that bacterial symbionts can protect their hosts from parasites and predators [50–52]. For example, the symbiont of *Paederus* beetles synthesizes a chemical toxin that beetles can use as a defense against predators [53]. Also, the symbionts of the ladybird *H. axyridis* produce pyrazines that have a function in defense behavior [54]. It would be interesting to investigate whether and which bacteria in prey cause immune responses in their predators.

## Conclusions

The high-quality whole genome assembly of the predatory ladybird *C. montrouzieri* provides insights into its diet adaptation, including the expansion or contraction of the gene families encoding chemosensors and digestive and detoxifying enzymes, and the enrichment of differentially expressed genes in pathways of nutrient metabolism when using unnatural diets. We highlight the potential role of immune enhancement and evolution of genes encoding immune effectors in diet adaptation of this species. This genomic study of a biological control agent is valuable for improving our basic understanding of its feeding habits, and may assist in improving its utilization in biological control.

## Methods

### DNA extraction, genome sequencing and assembly

DNA was extracted from the whole body of ten female adults of *C. montrouzieri*. These individuals were derived from a population that has been reared under laboratory conditions (27 ± 1 °C, 80 ± 1% relative humidity (RH) and a 14:10 (L:D) h photoperiod) at Sun Yat-sen University since 2005 [55]. Genomic DNA was extracted using the CTAB method [56]. The quality and concentration of the extracted genomic DNA were checked using 1% agarose gel electrophoresis and a Qubit fluorimeter (Invitrogen, Carlsbad, CA, USA). High-quality DNA was used for subsequent Nanopore and Illumina sequencing.

Approximately 15 μg of genomic DNA was used to generate Oxford Nanopore long reads, and the sequencing reaction was performed in a PromethION DNA sequencer (Oxford Nanopore, Oxford, UK). The raw data were then filtered to remove short sequence reads (< 5 kb) and reads with low-quality bases (Q30 < 90%) using Nanofilt v2.3.0 [57]. For assembly of Nanopore sequencing data, Canu v1.5 [58] was implemented to generate more accurate self-corrected reads with a corrected error rate of 0.05. Assembly was then performed by Wtdbg (https://github.com/ruanjue/wtdbg) with default settings. Racon v1.32 [59] was implemented to correct the assembly with Nanopore reads through two rounds with default settings. For further error correction, genomic DNA was also sequenced on the Illumina HiSeq X Ten platform (Illumina, San Diego, CA, USA). The Illumina sequenced data were filtered to remove reads with low-quality bases and adapters using Trimmomatic v0.36 [60] with default settings. Pilon v1.21 [61] was implemented to correct the Nanopore assembly with Illumina reads through three rounds with default settings.

### Gene prediction and functional annotation

First, the repetitive elements of the assembled genome were identified and masked. Repetitive elements of the assembled genome were classified into families with five rounds of RepeatModeler v2.0.1 analysis with default settings, followed by genome masking with RepeatMasker v4.1.0 (http://www.repeatmasker.org/) with default settings. Second, genes were automatically predicted based on our RNA-Seq data of *C. montrouzieri* in different life stages and diet treatments (SRA accession: SRR2971112, SRR2971116, SRR6981477, SRR8325176, SRR8325159). RNA-Seq reads were mapped to the assembled genome sequence using HISAT2 v2.1.0 [62]. Augustus 3.0.3 [63] was trained with the mapped data according to the Braker2 pipeline [24], and further used to predict genes in the genome sequences ab initio. BUSCO v4.1.2 [23] with the Insecta set of the OrthoDB v10.1 database was then used to assess the quality of the predicted gene set.

The protein sequences of the automatically predicted genes were subjected to similarity searches against the NCBI NR Hexapoda subset and UniProtKB Swiss-Prot databases using BLASTp with a cutoff E-value of 10^− 5^. Only the longest protein isoform for each gene was used as a query. Protein domains within genes were searched against the Pfam v32 database using InterProScan v5 [64] with a cutoff E-value of 10^− 5^. Sequences were also mapped to the GO database [65, 66] and KEGG reference pathways [67] using eggNOG-mapper [68] with a cutoff E-value of 10^− 5^.

In addition, specific genes with immune functions of beetles were identified based on searches against the databases UniProtKB/Swiss-Prot, Pfam v32 or KEGG pathway. These genes are classified into three major roles in immunity: recognition, signaling cascade and response [12, 42, 69]. Recognition genes included *c-type lectin* (containing the Pfam protein domain: ID:PF00059), *peptidoglycan recognition protein* (*PGRP*, identified by Swiss-Prot annotation) and *gram-negative binding protein* (*GNBP*, identified by Swiss-Prot annotation). Signaling cascade genes included those in the Toll and IMD pathway (KEGG: map 04624) and JAK/STAT pathway (KEGG: map 04630). Immune response genes included those encoding antimicrobial peptides, e.g., *attacin* (containing the Pfam domain: ID: PF03769), *defensin* (PF01097), *coleoptericin* (PF06286), *thaumatin* (PF00314) and *apolipophorin* (PF07464), and lysozymes, e.g., *c-type* and *i-type lysozyme* (identified by Swiss-Prot annotation). In addition, a putative antimicrobial gene, *cell wall hydrolase* (containing the Pfam domain: ID: PF07486), in ladybirds was also included in the analyses [70].

### Orthology search and gene family evolution

OrthoFinder v2 [71] was used to identify orthologous genes by retrieving the protein sequences of the *C. montrouzieri* and the other published Coleoptera genomes with default settings. A total of nine gene sets of Coleoptera genomes predicted from RNA-Seq data were used (Table 1), and their longest protein isoforms were extracted as input of OrthoFinder. Protein domains within genes were searched against the Pfam v32 database using InterProScan v5 with a cutoff E-value of 10^− 5^. Information of protein domain was subsequently assigned to the orthogroups using KinFin [72]. Furthermore, these orthogroups were used as input for CAFE v4.1 [30] to assess gene family contraction and expansion dynamics using the birth/death parameter (λ). The species tree used in CAFE was adapted from a recently published Coleoptera phylogeny [6]. In each branch, orthologous groups with *p*-values < 0.05 were considered significant expansions or contractions.

### Feeding experiment: diet-specific life history traits and transcriptome

Diet materials for *C. montrouzieri* were selected based on common protein sources used for insect diets. The selected protein sources covered whole bodies of seven invertebrate species, the eggs of three invertebrate species, two types of vertebrate products and one type of plant tissue (Table 3). Most solid materials were first dried in an oven (60 ± 1 °C) for 24 h and then ground to a powder using a kitchen blender. For materials that could not be directly fed to ladybirds, media were made with 2.5 g of protein source, 1.5 g of sucrose, 0.23 g of agar, and 19 mL of distilled water; sucrose was included as a feeding stimulant and agar as a thickener. A preliminary blank control treatment in which *C. montrouzieri* was offered the medium without a protein source from the 2nd instar onwards showed that none of the larvae developed to the 4th instar. We also set up a starvation treatment with no food or water provided by the 2nd instar onwards.

The developmental traits of *C. montrouzieri* were investigated from the 2nd instar onwards because we observed that some first instars died from non-nutritional factors (e.g., they were stuck in the medium). Before the treatments, all larvae were fed mealybugs in communal cultures. Thereafter, 52 to 137 2nd-instar larvae of *C. montrouzieri* (< 24 h after molting) derived from the population at Sun Yat-sen University [55] were placed individually in plastic Petri dishes (diameter: 5 cm, height: 2 cm) for the different diet treatments. All diets were offered ad libitum and replenished daily. The survival and development of *C. montrouzieri* larvae and pupae were monitored daily. Newly emerged adults were weighed. All feeding experiments were performed in a climatic chamber at 27 ± 1 °C with an 80 ± 1% relative humidity (RH) and a 14:10 (L:D) h photoperiod. A Kolmogorov-Smirnov test indicated that survival time of larvae and adult weight were normally distributed and therefore could be analyzed using a one-way analysis of variance (ANOVA). As a Levene test indicated homoscedasticity, the means were separated using Tukey tests. In all tests, *p* values below 0.05 were considered significant. All data were analyzed using SPSS 17.0 (SPSS Inc.).

Two 4th-instar larvae (< 24 h after molting) of *C. montrouzieri* from each diet treatment in the above life history experiment were randomly collected for transcriptome analysis. After ~ 12 h of starvation, the total RNA of each individual was extracted using TRIzol reagent (CWBIO, Beijing, China). RNA quality and quantity were determined using a Nanodrop 1000 spectrophotometer (Thermo Fisher Scientific, Wilmington, US). Only RNA samples with a 260/280 ratio from 1.8 to 2.0, a 260/230 ratio from 2.0 to 2.5 and an RNA integrity number (RIN) greater than 8.0 were used for sequencing. Sequencing was performed on the Illumina HiSeq 2500 platform, generating 2 × 125 bp reads. Adaptors and low-quality sequences were removed using the default settings for Trimmomatic v0.36.

Transcript assembly and abundance estimation were performed using the TopHat2 + Cufflinks method [73]. The coefficient of determination (*r*^2^) from Pearson’s correlation analysis was used to analyze the relationship of each sample pair based on fragments per kilobase of transcript per million mapped reads (FPKM) values. The regulation of gene expression was tested using Cuffdiff in Cufflinks, with a false discovery rate (FDR) < 0.05 for defining differentially expressed genes (DEGs). We used natural prey as a control to test for transcriptional regulation when ladybirds were fed unnatural diets. Thus, analyses of DEGs were performed only in the comparisons of the 12 factitious prey or artificial diet treatments against the MEALYBUG treatment. We investigated which GO terms and KEGG pathways the DEGs were involved in and evaluated statistical significance of GO and KEGG enrichment by hypergeometric distribution testing.

## Supplementary Information

**Additional file 1: Supplementary Figure S1.** Results of CAFE analysis inferring the size change of gene families in the genome of nine tested Coleoptera species. This summary tree shows the number of expanded (red) and contracted (green) families. The species’ ultrametric tree was adapted from Mckenna et al. [6]. Abbreviations of the tested species are defined in Table 1. **Table S3.** Comparison of life history traits of *Cryptolaemus montrouzieri* subjected to different diet treatments and starvation. Means (± SE) within a column followed by the same letter are not significantly different (*P* > 0.05). **Figure S2.** Heatmap of adjusted *P* values (Q) in Gene Ontology (GO) enrichment analysis for the transcriptome comparisons of alternative diets versus the natural prey of *C. montrouzieri* larvae. C: Cellular component; F: Molecular function; P: Biological process. The number of background genes of each GO term is shown in brackets. Enrichment with Q < 0.05 is marked with an asterisk.

**Additional file 2: Table S1.** Functional annotation of the gene set predicted from the genome of *Cryptolaemus montrouzieri*. **Table S2.** Information on the orthologous groups identified by OrthoFinder, number of genes in each genome/orthologous group and the *p*-value in the branch of *Cryptolaemus montrouzieri* estimated by CAFE. **Table S4.** Details of transcriptome profiling of *Cryptolaemus montrouzieri* under different diet treatments. Expression levels of genes of the 12 factitious prey or artificial diet treatments were compared to those of the mealybug treatment. Values of fragments per kilobase of transcript per million mapped reads (FPKM) of each gene/treatment and log2(fold change) and adjusted *p* of each gene/comparison are shown.

## Data Availability

The raw and assembled sequenced data of *C. montrouzieri* were deposited in the NCBI BioProject: PRJNA626074. The RNA-Seq raw data of different diet treatments of *C. montrouzieri* were deposited in the Sequence Read Archive (SRA) repository of the NCBI under accession Nos. SRR8325159 - SRR8325188 of BioProject PRJNA509782.
