# Supplementary material for: Genomic insight into diet adaptation in the biological control agent Cryptolaemus montrouzieri
Source: BMC Genomics. 2021 Feb 25;22:135. doi: 10.1186/s12864-021-07442-3 (PMC7905881; doi:10.1186/s12864-021-07442-3)
Supplement: Supplementary file 1 — Additional file 1: Supplementary Figure S1. Results of CAFE analysis inferring the size change of gene families in the genome of nine tested Coleoptera species. This summary tree shows the number of expanded (red) and contracted (green) families. The species’ ultrametric tree was adapted from Mckenna et al. [6]. Abbreviations of the tested species are defined in Table 1. Table S3. Comparison of life history traits of Cryptolaemus montrouzieri subjected to different diet treatments and starvation. Means (± SE) within a column followed by the same letter are not significantly different (P > 0.05). Figure S2. Heatmap of adjusted P values (Q) in Gene Ontology (GO) enrichment analysis for the transcriptome comparisons of alternative diets versus the natural prey of C. montrouzieri larvae. C: Cellular component; F: Molecular function; P: Biological process. The number of background genes of each GO term is shown in brackets. Enrichment with Q < 0.05 is marked with an asterisk. [file 12864_2021_7442_MOESM1_ESM.docx]

Supplementary file 1: Figure S1, Figure S2, Table S3

Genomic insight into diet adaptation in the biological control agent *Cryptolaemus montrouzieri*

Hao-Sen Li^1^, Yu-Hao Huang^1^, Mei-Lan Chen^1,2^, Zhan Ren^1^, Bo-Yuan Qiu^1^, Patrick De Clercq^3^, Gerald Heckel^4^, Hong Pang^1^*

1 State Key Laboratory of Biocontrol, School of Life Sciences / School of Ecology, Sun Yat‐sen University, Guangzhou, Guangdong, China

2 School of Environment and Life Science, Nanning Normal University, Nanning 530001, China

3 Department of Plants and Crops, Faculty of Bioscience Engineering, Ghent University, Ghent, Belgium

4 Institute of Ecology and Evolution, University of Bern, Bern, Switzerland

*Author for Correspondence: lsshpang@mail.sysu.edu.cn


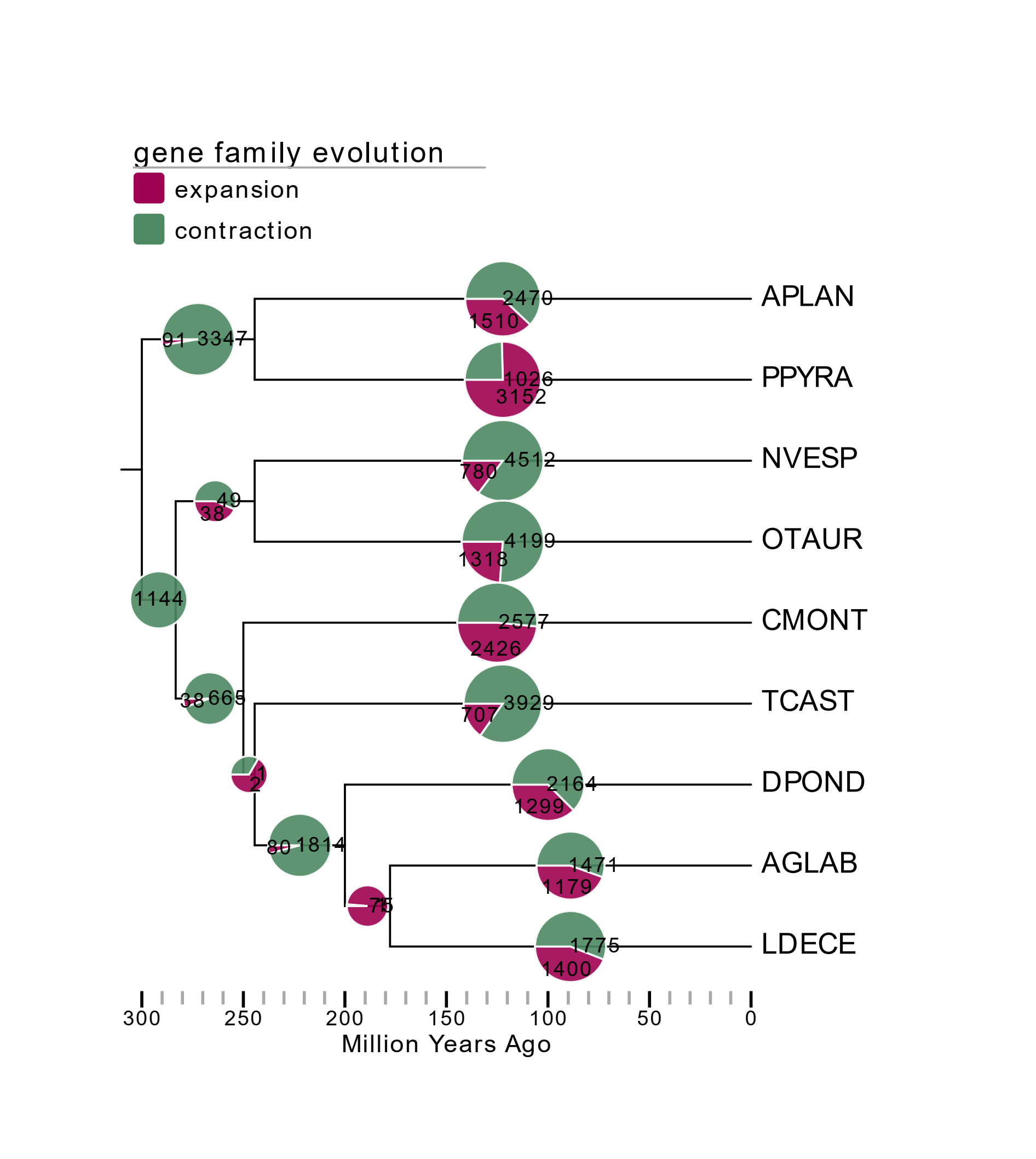


Figure S1


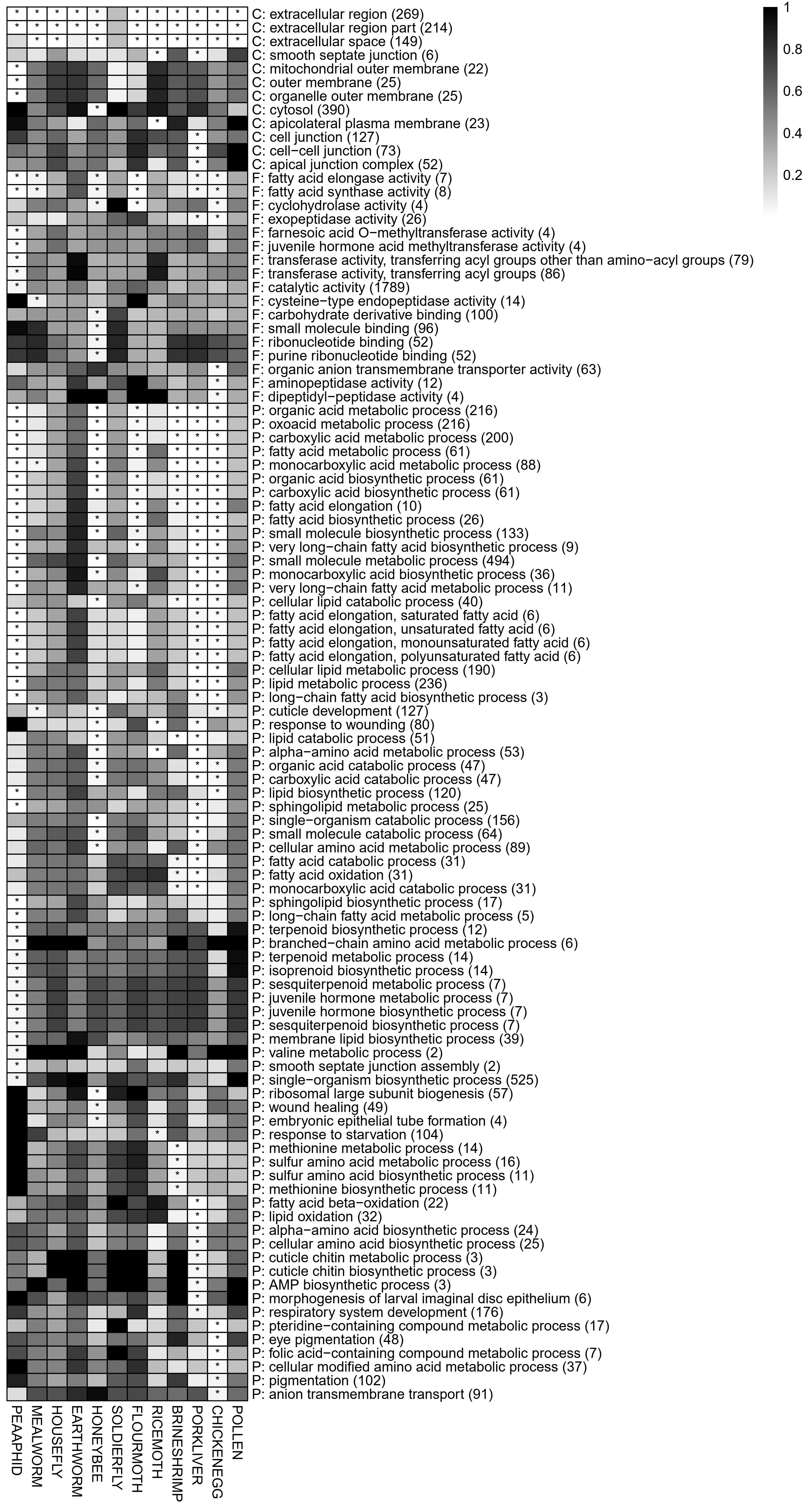


Figure S2


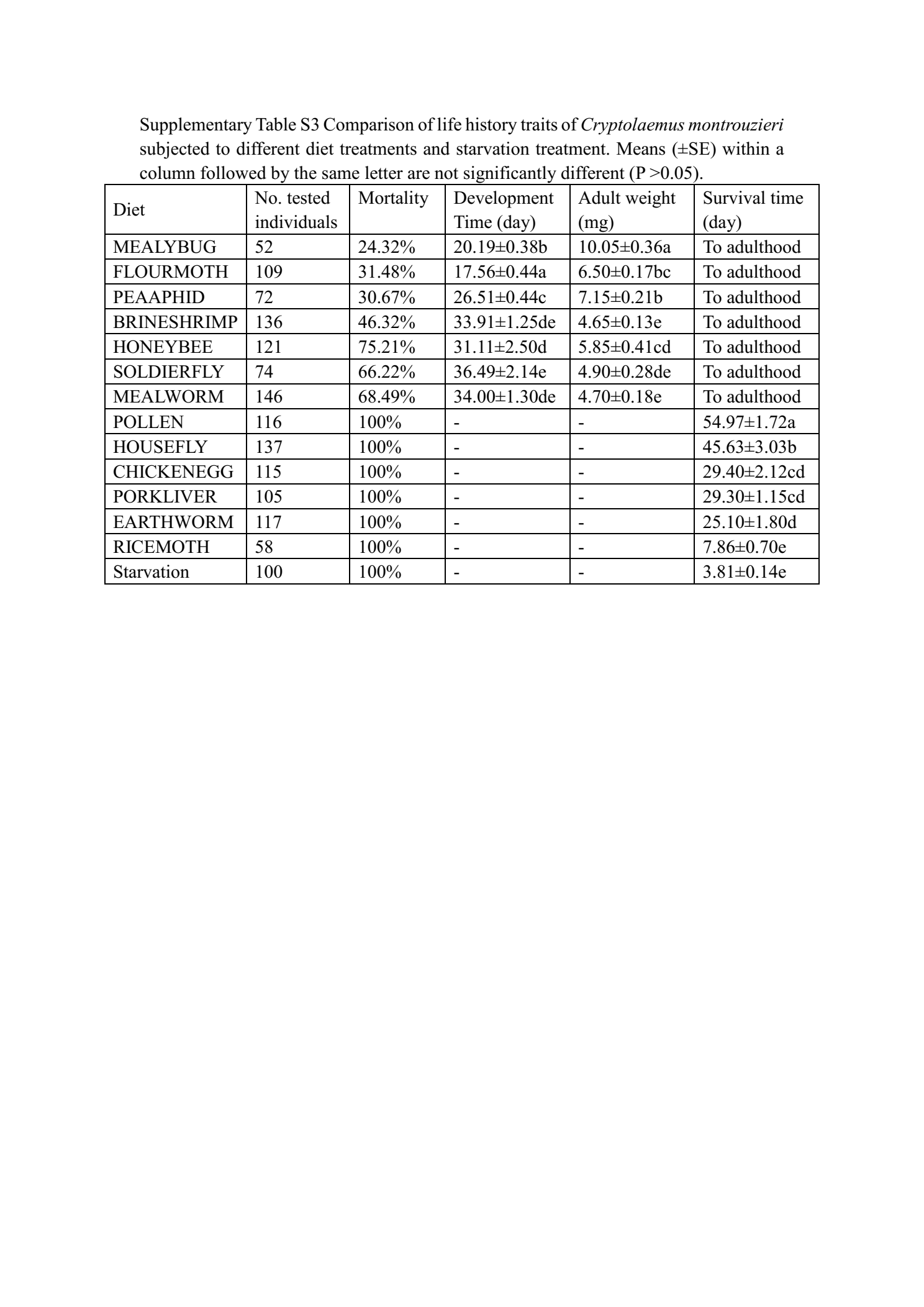


Table S3
